# The Recent Outbreaks and a Great Need

**Published:** 1919-12-20

**Authors:** 


### DIPHTHERIA AND ANTITOXIN.

### The Recent Outbreaks and a Great Need.

During the last few weeks public attention has been drawn by the Press, as well as by the obvious incidence of the disease, to the fact that London—and, indeed, many parts of the country—are suffering from a somewhat severe outbreak of diphtheria. The value of this publicity is undoubted, but requires not to be written up on alarmist lines. We must remember that season has a marked influence on the manifestation, and particularly upon the mortality from the disease. Increased prevalences quite commonly commence in September, reach their highest point in October and November, and then slowly subside during the following months. Another point is that, according to Newsholme, diphtheria tends to become more widely epidemic in those years of deficient rainfall. Thus the present situation is largely in accordance with past experience, and the somewhat high figures for the month do not necessarily give rise to anxiety. Nevertheless there is one point which should be noted. Until the last few years diphtheria was regarded as being to a far greater extent a rural rather than an urban disease. During the last thirty, however, the mortality has progressively increased in London and several other large cities, and, to quote official figures, we find that in 1881 in London the death-rate from diphtheria was 0.17 per 1,000, the average of the ten years 1871-80 being 0.12 per 1,000. Then the average for the decennium 1881-90 was 0.26 per 1,000, and for the decennium 1891-1900 0.50 per 1,000. Since about 1899, however, it is found that there has been a steady decline in diphtheria mortality. With the first figures the incidence runs approximately parallel with the number of deaths, but midway in the last period—*i.e.*, 1890 to 1899—the two diverge. We still have the greater prevalence, but at this time the use of prepared anti-toxic serum was adopted, and came into more or less general and regular use in 1894, with the well-known effect upon the mortality rate. No more striking example can be given than the fact that the case mortality in the Metropolitan Asylums Board's hospitals was, in the pre-antitoxin days, 30.3 per cent., and is now under 9 per cent. generally, while among those treated on the first day of the disease only 3 per cent. die.

Now we know that in order to facilitate the early administration of the serum, a great many local authorities have kept a stock at their public offices, and under the advice and sanction of the Local Government Board have supplied it to practitioners at cost price or gratuitously in the case of poor patients. The results have been invaluable, from the obviously high importance of giving a large initial dose early in the disease, and also from the fact that the antitoxin possesses great prophylactic worth and may be used with advantage in doses of at least 1,000 units for the protection of children who are or have been exposed to the risks of infection.

At the moment we are at a phase of most extensive reconstruction in the country's general medical organisation and one of almost unparalleled possibilities. Now is the time that we must make the utmost of such advantages as the invention of diphtheria antitoxin has placed in our hands. Of the disease, its complication, its widespread incidence, and the dire remote effects it can produce there is no need here to speak in detail; the matter has been fully discussed in a famous contemporary, where both the lay and professional publics cannot have failed to appreciate its importance.

Following, therefore, in the wake of this necessary reminder comes most appropriately a letter in the *Times* from a member of a hospital committee, which may well be quoted:

> For antitoxin alone each case costs my hard-up hospital from 20s. to 25s. The cost is prohibitive to many general practitioners, especially those who practice among the "new poor." May I urge, through your columns, that the State should manufacture and supply this serum free of charge, both to hospitals and general practitioners? I am not enamoured of subsidies or doles, but the direct saving of life justifies departure from economic principles. It is both painful and irritating to see a patient choking to death for want of timely and adequate treatment by a well-known and sure remedy. The other day I saw a small boy just dying. He was not brought to the hospital till the sixth or seventh day of the disease. In treatment at his home he had been given a wholly inadequate dose, and the serum had been given by the mouth instead of subcutaneously. Nurses, of course, contract the disease, and will continue to do so. They cheerfully take the risk, as they take any other risk incident to their self-sacrificing profession. The last time I went through our wards I found three nurses down with diphtheria. In bygone days I have seen valuable and devoted nurses die from the disease, caught in the course of their work. Now they get prompt treatment. Since we have had antitoxin, and learned how to use it, I am glad to say I have never seen a fatal case among our nursing staff.

One quotes thus fully, in that the writer's experience corresponds with and typifies that in practically every hospital. The institutions themselves may obtain an adequate supply of the serum even though it costs them many pounds, but it is an everyday happening that the cases which reach them have not received the serum at the earliest possible moment, namely coincident with their first encounter with a medical man. It is an urgent necessity that this therapeutic agent should be, if not available free, at least available at such a price and in such quantity that it is easily at hand for those dealing with the masses of the country. The methods of preventing spread of the disease itself form a quite separate consideration. General improvements in hygiene and sanitation are doubtless progressing towards a gradually increased limitation, but at present we must be prepared for the customary high rate of infection. Therefore, to save the country from the effects of this "mother of disease," it is the curative agent which is needed

**Diphtheria and Antitoxin**—(*continued*).

in free and ever-increasing availability. And let us too remember that the lessened case mortality, which has resulted from the use of antitoxin, is not attributable to any gradual attenuation of the virus, for it is a fact that in several home and continental towns, whilst the local incidence of the disease has remained unchanged among people in the same community, there is reduction in the case mortality only among those who have had the freest use of antitoxin made for them.

It is true that the preparation of the sera is a highly specialised process, but at the same time one particularly amenable to reduction of costs by the process of mass production. During the war ample evidence has been forthcoming of the readiness with which supplies of laboratory-made agents could be turned out if organised effort were made, and it is to be hoped that our new health organisation will share no effort to improve by every means possible, the supply of diphtheria antitoxin to the poorer public.

